# Prevalence and Characterisation of Severe Left Ventricular Hypertrophy Diagnosed by Echocardiography in Hypertensive Patients

**DOI:** 10.3390/jcm12010228

**Published:** 2022-12-28

**Authors:** Anett Apitz, Thenral Socrates, Thilo Burkard, Michael Mayr, Annina S. Vischer

**Affiliations:** 1 Medical Outpatient Department and Hypertension Clinic, ESH Hypertension Centre of Excellence, University Hospital Basel, 4031 Basel, Switzerland; 2 Department of Cardiology, University Hospital Basel, 4031 Basel, Switzerland; 3 Faculty of Medicine, University of Basel, 4056 Basel, Switzerland

**Keywords:** arterial hypertension, blood pressure, left ventricular hypertrophy, hypertension-mediated organ damage, echocardiography, electrocardiography

## Abstract

Background: Arterial hypertension (AHT) is the leading preventable cause of death worldwide. Left ventricular hypertrophy (LVH) is one of the most important prognostic markers in hypertension and a predictor for mortality. The goals of this study were to examine the prevalence of LVH detected by echocardiography in patients with AHT and to describe patients with severe LVH. Methods: This is a retrospective monocentric study including patients treated at a tertiary hypertension clinic. Echocardiographic data were taken from written reports from our hospital’s echocardiography laboratories. We compared patients with severe LVH (septum thickness ≥ 15 mm) with patients with normal left ventricular (LV) geometry and with patients with concentric or eccentric hypertrophy regarding age, gender, comorbidities, medication, duration of hypertension, blood pressure (BP) and ECG changes at time of echocardiography. Results: Twenty-nine patients (7.3%) out of four hundred patients showed severe LVH and one hundred and eighty-nine (47.3%) a normal geometry. In comparison to patients with normal geometry, patients with severe LVH were more likely to be male, older, and with more uncontrolled BP, especially regarding asleep values, multi-drug antihypertensive treatment and comorbidities. In comparison to patients with concentric or eccentric hypertrophy, patients with severe LVH had a significantly higher diastolic BP in the 24 h mean, awake and asleep values. A positive Sokolow-Lyon index did not predict LVH. However, patients with severe LVH were more likely to have T-wave-inversions V4–V6 in at least one lead. Conclusions: More than half of the patients with AHT have an abnormal geometry in our study (52.5%) and 7.3% a severe LVH. Patients with severe LVH have more often an uncontrolled AHT than patients with a normal LV geometry, despite more antihypertensive treatment. The Sokolow-Lyon index seems to be insufficient to detect LVH.

## 1. Introduction

Arterial hypertension (AHT) is the leading preventable cause of premature death worldwide [1]. Left ventricular hypertrophy (LVH) is one of the most important prognostic markers in AHT and a predictor of mortality [2]. Blood pressure (BP)-lowering treatment reduces LVH [3] and the rate of a composite cardiovascular endpoint [4]. 

LVH can be caused not only from AHT, but also from, to name a few causes, aortic valve stenosis [5,6] and hypertrophic cardiomyopathy (HCM). The differentiation between hypertension-mediated LVH and HCM can be difficult in the daily routine, but essential for the treatment. Cardiac magnetic resonance imaging (CMR) can be especially helpful for the discrimination between HCM and hypertensive heart disease (HHD) [7].

The 1988 Framingham Heart Study detected LVH in 16% of the men and in 19% of the women in the general population with or without AHT by echocardiography. Especially in the youngest age groups in this study, LVH was more prevalent in men, whereas, in older subjects, it was more prevalent in females [8]. These echocardiographic data are more than 30 years old. Since then, the definitions for AHT, the indications for performing echocardiography and the technical means for diagnosis have changed. Population-based studies, including the Atherosclerotic Risk in Communities and the PAMELA studies, have examined the prevalence of the different geometry patterns, and this topic was also covered in a review article [9,10,11,12]. However, none of these studies analysed the amount of LVH, and there is no information on the prevalence of severe LVH and the characteristics of these patients.

Lehtonen et al. only applied electrocardiography, a less sensitive method, which detected a positive Sokolow-Lyon index as a marker of LVH in 11–13% patients with an AHT [13]. In a very selective cohort of patients with known AHT and normal electrocardiogram (ECG), the prevalence of LVH was reported as 8% [14].

The aim of this study is to examine the prevalence of LVH in patients with AHT by echocardiography and, specifically, to describe patients with severe LVH in AHT.

## 2. Materials and Methods

### 2.1. Study Population

This is a retrospective monocentric cross-sectional study with patients with AHT treated at a tertiary hypertension clinic, treated from June 2016 to December 2019 in the Medical Outpatient Department of the University Hospital in Basel, Switzerland. We included all consecutive patients with known AHT from our hypertension clinic receiving an echocardiography before or during the treatment. Patients without an echocardiographic examination were excluded.

### 2.2. Documented Parameters

#### 2.2.1. Echocardiography

Transthoracic echocardiographic data were obtained from the report of the echocardiography laboratory of our hospital. The echocardiography was mainly performed during the initial consultation. When no report from our hospital was available, the reports from the referring cardiologists were used. Reports from echocardiography were scrutinised for ejection fraction (LVEF in %), anteroposterior left atrial diameter (LA in mm), left atrial volume index (LAVI in ml/m^2^), left ventricular mass index (LVMI in g/m^2^), septal thickness (mm), posterior wall thickness (mm) and relative wall thickness (calculated by the formula (2 × posterior wall thickness)/(left ventricular internal diameter at end-diastole)). Echocardiographic measurements were performed according to current guidelines [15]. Only few patients had more than one echocardiographic examination. When multiple examinations were available, the most recent evaluation was used. 

Only patients with results on septal thickness, LVMI and relative wall thickness were included. 

**Figure 1:**
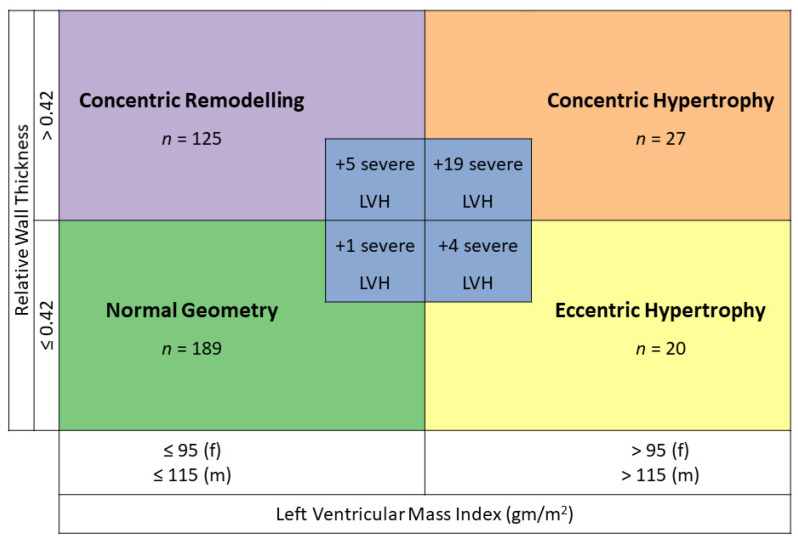
Definition and distribution of left ventricular geometry (adapted after [11]). LVH: left ventricular hypertrophy.

Left ventricular (LV) geometry was classified according to current American Society of Echocardiography guidelines as follows: normal, concentric remodelling, concentric hypertrophy, eccentric hypertrophy and severe LVH. Patients with a septum thickness of ≥ 15 mm were regarded as having severe LVH according to the HCM guidelines of ESC 2014 [7]. A normal LV geometry was defined as a relative wall thickness ≤ 0.42 and a LVMI ≤ 115 g/m^2^ for men and ≤ 95 g/m^2^ for women (Figure 1). Concentric remodelling was defined as a relative wall thickness > 0.42 and a LVMI ≤ 115 g/m^2^ and ≤ 95 g/m^2^ for men and women, respectively. Concentric hypertrophy was defined as a relative wall thickness > 0.42 and a LVMI >115 g/m^2^ and > 95 g/m^2^ for men and women, respectively. Eccentric hypertrophy was defined as a relative wall thickness ≤ 0.42 and a LVMI ≥ 115 g/m^2^ and ≥ 95 g/m^2^ for men and women, respectively. Hypertensive heart disease was labelled in case of LVMI ≥ 115 g/m^2^ and ≥ 95 g/m^2^ for men and women, respectively, thus concentric or eccentric hypertrophy, according to the ESC/ESH hypertension guidelines [16]. 

#### 2.2.2. Blood Pressure Measurements

We used the 24 h-ambulatory BP measurement (ABPM) chronologically closest to the echocardiographic examination. Time in bed according to the patient diary was used to define awake and asleep values. Furthermore, we documented the 24 h mean value. We labelled AHT as uncontrolled if the 24 h mean value was ≥ 130 mmHg systolic and/or ≥ 80 mmHg diastolic, the awake value ≥ 135 mmHg systolic and/or ≥ 85 mmHg diastolic or the asleep value ≥ 120 mmHg systolic and/or ≥ 70 mmHg diastolic.

#### 2.2.3. Electrocardiogram

The ECGs from the time of the echocardiography were analysed by an experienced internist (AA). We documented the Sokolow-Lyon index (SV1 + RV5 > 3.5 mV) [17], flat or negative T waves and any ST-Depression (>1 mm).

#### 2.2.4. Medication

The medications were obtained from the patient records at the time of the echocardiographic examination. We documented the medication classes, i.e., angiotensin converting enzyme inhibitors (ACE), angiotensin 1 receptor antagonist (ARB), calcium channel blockers (CCB), diuretics, beta blockers and any other antihypertensive medications. Furthermore, we documented if they had a mono-, dual- or triple therapy. 

#### 2.2.5. Comorbidities and Risk Factors

Comorbidities and other cardiovascular risk factors were assessed from the clinic records. We documented the BMI, smoking status, HbA1c, estimated glomerular filtration rate (eGFR), presence of albuminuria, history of coronary heart disease, aortic aneurysm, peripheral arterial disease or stroke/transient ischemic attack (TIA). 

#### 2.2.6. Blood Tests

We documented HbA1c (%) and estimated glomerular filtration rate (ml/min) and albuminuria (albumin–creatinine ratio in mg/mmol), albuminuria as target organ damage, defined by albumin–creatinine ratio ≥ 3.4 mg/mmol, analysed during regular clinical practice. In case of multiple assessments, we used the one chronologically closest to the echocardiography.

### 2.3. Formation of Groups 

Patients with severe LVH formed a separate group, regardless of their global LV geometry. All remaining patients were separated into the following groups according to their LV geometry: normal LV geometry, concentric remodelling, concentric hypertrophy and eccentric hypertrophy (see also Figure 1). 

To compare patients with severe LVH with patients without signs of hypertensive heart disease, we compared them with patients with normal LV geometry. This group was labelled “no LVH”. To compare patients with severe LVH with patients with a hypertensive heart disease, we combined patients with a concentric hypertrophy and patients with an eccentric hypertrophy to form the comparison group. This group was labelled “LVH”. 

### 2.4. Statistical Analysis

Continuous data were reported as mean ± standard deviation (SD) if normally distributed or otherwise as median (interquartile range (IQR)). The Shapiro–Wilk test was used to test for normality. We tested for between-group differences via an independent sample *t*-test/Mann–Whitney U test and Fisher’s exact rest, as applicable. To test for between-group differences between multiple groups, we applied a Kruskal–Wallis test and Fisher’s exact test, as applicable. A *p*-value < 0.05 was regarded as statistically significant. 

### 2.5. Ethical Approval

This trial was approved by the local ethics committee (EKNZ number: 2019-01537) and was compliant with the Declaration of Helsinki. All patients had completed a general informed consent to assess their data for retrospective research purposes. Patients without such consent were excluded.

## 3. Results

### 3.1. Baseline Characteristics

The study population included 422 patients. Due to insufficient echocardiography documentation or image quality, 22 patients had to be excluded, resulting in a final study population of 400 patients. One hundred and seventy-seven (44.3%) patients were women. The median age of the overall cohort was 56 years (IQR 43–70 years). One hundred and thirty patients (37.2%) had a secondary form of AHT. The median of the systolic and diastolic 24 h mean, awake and asleep BP was not in the target range (24 h mean ambulatory BP (ABP) ≥ 130/80 mmHg and/or awake ABP ≥135/85 mmHg and/or asleep ABP ≥ 120/70 mmHg) [18]. Complete baseline characteristics are shown in Table 1. 

### 3.2. Prevalence of Left Ventricular Hypertrophy

Both LVMI and relative wall thickness were available in 400 patients. Twenty percent of our patients (80 patients) had a concentric or eccentric LVH, thus a hypertensive heart disease related to the geometry. One hundred and ninety patients (47.5%) had a normal geometry, one hundred and thirty (32.5%) a concentric remodelling, fifty-six (14.0%) a concentric hypertrophy and twenty-four (6.0%) an eccentric hypertrophy (Figure 1). Overall, more than half of our patients (52.5%) with AHT had an abnormal geometry. 

**Figure 2:**
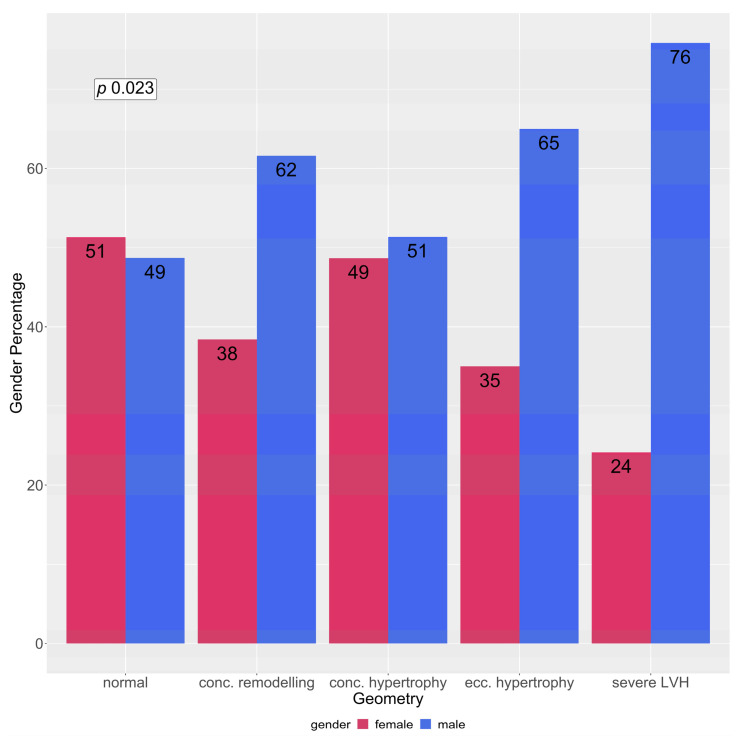
Left ventricular geometry according to gender. Red corresponds to female, blue to male. LVH: left ventricular hypertrophy. Numbers in columns refer to percentage of gender within geometry group. *p*-value calculated across all geometry groups using a Fisher’s exact test.

We identified 29 patients (7.3%) who had a severe LVH in echocardiography. A severe LVH could be found in all geometry forms. If geometry was classified according to the guidelines in those patients with severe LVH, 1 (3.4%) classified as normal, 5 (17.2%) as concentric remodelling, 19 (65.5%) as concentric hypertrophy and 4 (13.8%) as eccentric hypertrophy (Figure 1). The percentage of men was significantly higher in the group with severe LVH than in the group with normal geometry (75.9% vs. 48.7%, *p* value 0.009). The prevalence of LVH in patients with an AHT according to gender is shown in Figure 2.

### 3.3. Severe Left Ventricular Hypertrophy vs. No Left Ventricular Hypertrophy and vs. Left Ventricular Hypertrophy

There were 29 patients with severe LVH and 189 patients without LVH. The differences between patients with severe LVH and patients without LVH are shown in Table 2.

Patients with severe LVH were excluded from the other groups of LV geometry.

There was a significant male predominance in patients with a severe LVH in comparison to patients without LVH (75.9% vs. 48.7%, *p*-value 0.009). Patients with a severe LVH were significantly older (64.9 years) than those without LVH (50.4 years, *p*-value 0.001). Secondary forms of AHT were significantly more common in the group with the severe LVH than in the group without LVH (60.0% vs. 29.5%, *p*-value 0.005).

Patients with a severe LVH had a significantly higher systolic and diastolic BP regarding the 24 h mean, awake and asleep ABPM values in comparison to patients without LVH (Table 2). 

Patients with a severe LVH had significantly more often a coronary heart disease (37.9% vs. 19%, *p*-value 0.029) than patients without LVH. Patients with a severe LVH were also more likely to have a lower eGFR (eGFR mean 59.0 mL/min vs. 93.0 mL/min, *p*-value < 0.0005) and albuminuria > 3.4 mg/mmol (58.8% vs. 12.5%, *p*-value < 0.0005) as comorbidities compared to patients without LVH.

Significantly more people with a severe LVH had a therapy with diuretics (65.5% vs. 19.7%, *p*-value < 0.0005) and an antihypertensive triple therapy (44.8% vs. 14.3%, *p*-value < 0.0005) than patients without LVH. There was no difference in therapy with ACE inhibitors, ARB or calcium channel inhibitors between patients with a severe LVH and patients without LVH.

Though there was a trend, there were no significant differences regarding a positive Sokolow-Lyon index between the two groups, 11.1% in the group with severe LVH und 2.4% in the group without LVH (*p*-value 0.056). In contrast, we found significantly more T-wave changes, i.e., a negative T-wave in at least one lead V4-V6, in the ECG of patients with a severe LVH compared to patients without LVH (18% vs. 4.1%, *p*-value 0.014). However, 2 of the 29 patients with severe LVH did not have an ECG.

The Sokolow-Lyon index had a sensitivity of 11%, a positive predictive value of 19%, a specificity of 96% and negative predictive value of 93% for severe LVH in our study.

The differences between patients with severe LVH and patients with LVH but a septal thickness < 15 mm are shown in Table 2. There were 57 patients with non-severe LVH. Especially diastolic BP was significantly higher in the 24 h mean, awake and asleep values in the group with severe LVH in comparison to the patients with non-severe LVH. There were no significant differences in comorbidities, including secondary forms of AHT and antihypertensive medication, between the patients with severe LVH and patients with non-severe LVH. 

### 3.4. Gender Differences Regarding Blood Pressure across All Geometries 

**Figure 3:**
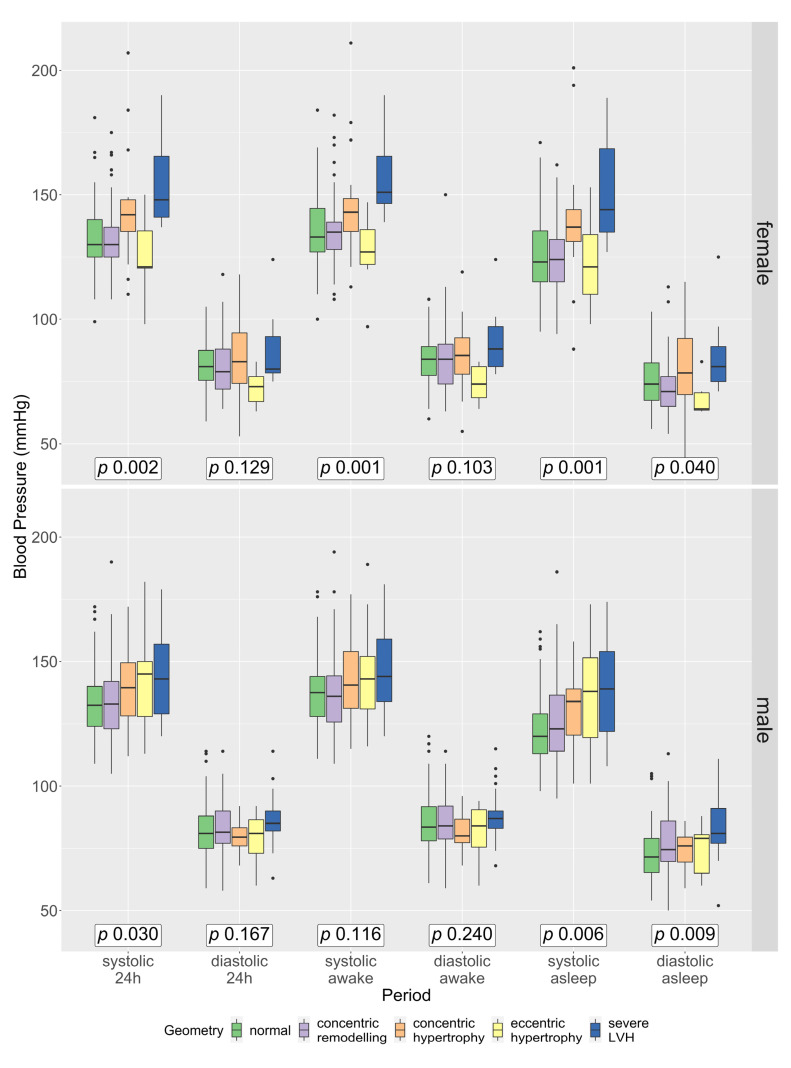
Systolic and diastolic blood pressure in the 24 h mean, awake and asleep values according to LV geometry and gender. Green: normal geometry, lilac: concentric remodelling, orange: concentric hypertrophy, yellow: eccentric hypertrophy, blue: severe LVH. *p*-values calculated with a Kruskal–Wallis test across all geometries per gender and per BP period.

Women with a severe LVH had significantly higher systolic 24 h mean, awake and asleep ABPM values than women with all other geometries; women with a severe LVH also had higher diastolic asleep values, with a trend for higher diastolic 24 h mean and awake values, although post hoc analyses support only the systolic findings (Figure S1). In men, however, this association was less pronounced and visually only observable in systolic values (Figure 3). Women with eccentric hypertrophy showed a trend towards lower systolic and lower diastolic 24 h mean, awake and asleep ABPM values than the other groups, whereas, in men, there was no difference between the ABPM values in those groups (Figure 3). Post hoc analyses in men showed only significant differences for systolic and diastolic asleep values between patients with a severe LVH and patients with a normal geometry (Figure S1). Patients with a severe LVH showed significantly more uncontrolled systolic and diastolic BP at night than those with a normal geometry (Figure S2). There was no association between the duration of AHT and the presence of severe LVH.

### 3.5. Differences Regarding Blood Pressure across All Geometries 

**Figure 4:**
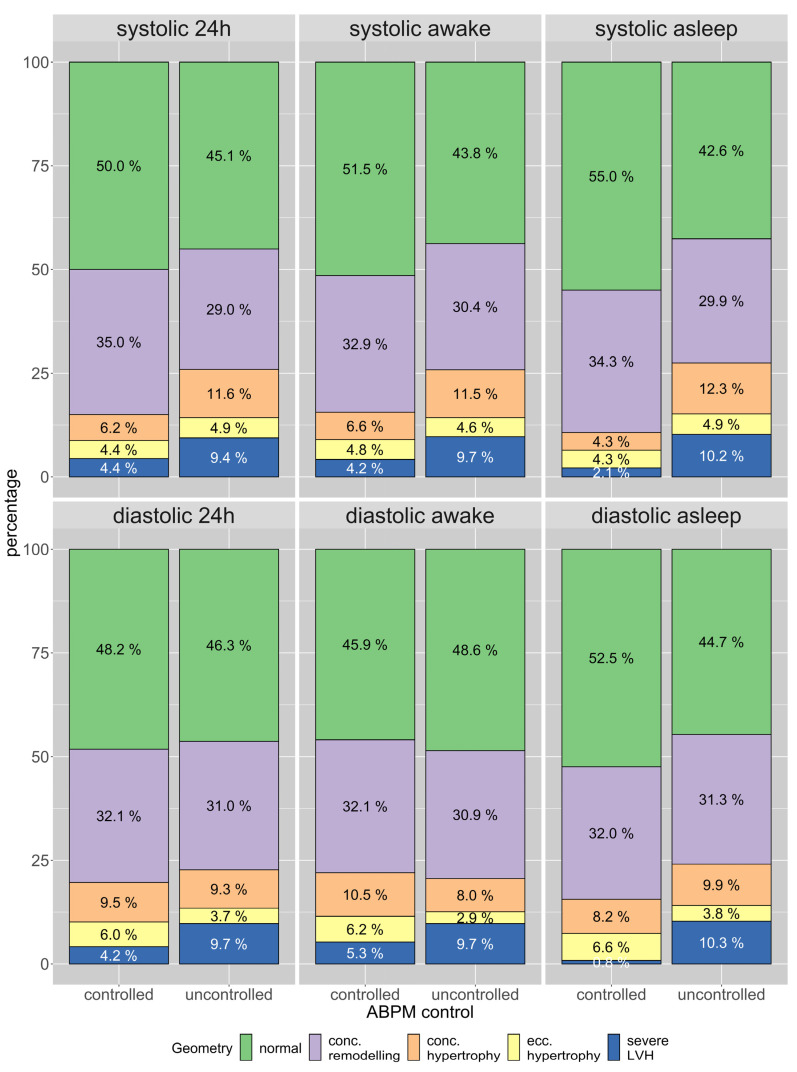
Distribution of LV geometries in patients with controlled and uncontrolled systolic and diastolic ambulatory blood pressure measurements (ABPM) in the 24 h mean, awake and asleep values. Green: normal geometry, lilac: concentric remodelling, orange: concentric hypertrophy, yellow: eccentric hypertrophy, blue: severe LVH.

The predominant geometry across patients with and without controlled BP was a normal geometry. Patients with uncontrolled systolic or diastolic BP had more frequently a severe LVH. Patients with an uncontrolled systolic BP also had more frequently a concentric hypertrophy. This effect was most apparent in the asleep BP values (Figure 4). 

**Figure 5:**
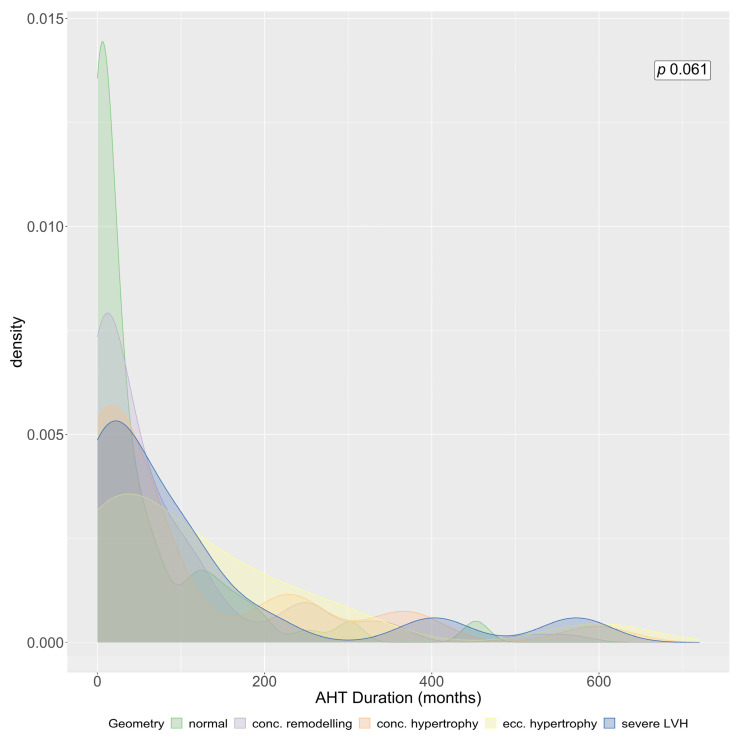
Density plot of arterial hypertension (AHT) duration for each LV geometry: Green: normal geometry, lilac: concentric remodelling, orange: concentric hypertrophy, yellow: eccentric hypertrophy, blue: severe LVH. *p*-value calculated with a Kruskal–Wallis test.

There were no significant differences regarding the AHT duration between patients with and without severe LVH (Table 2, Figure 5), although most patients without LVH had a short AHT duration documented, whereas more patients with a more severe form of LVH had a longer AHT duration. 

## 4. Discussion

In our contemporary population of patients referred to a hypertension clinic of a tertiary hospital, 7.25% of all included patients with AHT had a severe LVH and 14.25% a concentric or eccentric LVH, i.e., a hypertensive heart disease based on the LV geometry. Patients with a severe LVH were more likely male and older than patients with a normal LV geometry. Furthermore, they had a higher BP, more antihypertensive medications and comorbidities. These variables can be regarded as markers of more severe forms of AHT. However, it is unclear how adherence was in patients who had to take many medications. Interestingly, there was no statistical significance regarding the duration of AHT, although, numerically, the duration was longer in patients with severe LVH.

Many included patients were relatively recently diagnosed with AHT (median 23 months). However, it is never certain how long the AHT existed before diagnosis. Patients with severe LVH had a tendency to have had AHT longer, although since this was possibly due to the small number of patients, this result was not significant.

Patients with a severe LVH had significantly higher systolic and diastolic BP values than patients with a normal LV geometry. In comparison to patients with a concentric or eccentric hypertrophy, only the diastolic values were significantly higher in patients with severe LVH. Furthermore, patients with a severe LVH had significantly more often uncontrolled systolic and diastolic 24 h mean BP values, again only for diastolic values in comparison to patients with concentric or eccentric hypertrophy. Considering that the severe LVH is most likely a consequence of the higher BP values, this seems, at first glance, to be a contradiction to the well-known data of the Dublin outcome study [19], which showed that the systolic asleep values in particular are most important for prognostic purposes. One key to this may be in the gender distribution. In the Dublin outcome study, 55% of the participants were women, as were 44% of the participants experiencing a cardiovascular death. In our study, fewer women (44%) were included, and only 24% of those patients with a severe LVH were female. Referring to Figure 3 and Figure S1, women with severe LVH have higher systolic and diastolic values, whereas, in men, it is numerically only the diastolic values. Therefore, the male dominance in our study could have caused the focus of the diastolic value in our data for the prediction of LVH.

With regard to other target organ damage, we could only determine statistical significance with regard to coronary heart disease, albuminuria and eGFR between the patients with severe LVH and patients with normal LV geometry. It has been shown that proteinuria is a very good prognostic parameter to predict long-term cardiovascular events in hypertensive patients [20]. Our study population was probably too small, or the prevalence of stroke, transient ischemic attack or peripheral arterial disease were too low, to find statistical significance. On the other hand, there were many patients who were recently diagnosed with AHT, so they may not have had any cardiovascular events yet [21].

Surprisingly, only 11% of the patients with a severe LVH and, in contrast, 4 of patients (2.4%) with normal left ventricular geometry had a positive Sokolow-Lyon index, which is recommended in the ESC guidelines 2018 for screening of hypertension-mediated organ damage (HMOD) [16]. Considering its low sensitivity of 11%, the use of ECG for the detection of LVH should be questioned, although the specificity is high with 96%. Lehtonen et al. reported in their study on the prevalence and prognosis of ECG abnormalities in normotensive and hypertensive individuals [13] a significant increase of ST/T changes in participants with a Grade 2 AHT in comparison to normotensive participants. Overall, positive Sokolow-Lyon indices were more prevalent in this study than in ours, possibly due to the higher number of patients with AHT included (*n* = 1497 vs. *n* = 400 in our study). Furthermore, the true prevalence of LVH in this cohort is not known. The study on the performance of Sokolow-Lyon index in detection of echocardiographically diagnosed LVH in a normal Eastern German population shows that the correlation of the Sokolow-Lyon index and echocardiographic parameters of LVH is weak. Sensitivity of the Sokolow-Lyon index was 5% and the specificity 95% in this study [22] similar to our study (sensitivity 11% and specificity 96%). It is therefore questionable whether the Sokolow-Lyon index is suitable for diagnosing LVH.

Interestingly, patients with severe LVH had significantly more negative T waves (18% vs. 4%) in the ECG in the leads representing the anterolateral wall, resulting in a sensitivity of 19% and a specificity of 92%. This ECG change appear to be a more sensitive, though less specific parameter, in comparison to the Sokolow-Lyon index, for the LVH in the ECG in our patient population. 

Repolarization abnormalities are very common in patients with HCM [23] and can be a sign of a sarcomeric form of the disease. HCM is defined by the presence of increased LV wall thickness over 15 mm, which is not solely explained by abnormal loading conditions [7]. HCM cannot be diagnosed with certainty on echocardiography alone [24]. CMR markers such as native myocardial T1 mapping, late gadolinium enhancement or global longitudinal strain might be helpful; however, CMR was too rarely ordered in our study to be able to make a statement in this regard [25]. Further factors potentially helping in the differentiation between severe LVH due to AHT and HCM might be age, location of the hypertrophy and remodelling over time or the MESA index; however, these parameters were not studied in our cohort.

Limitations of our study are the small study population, the selection of patients of a dedicated hypertension clinic and the low number of CMRs ordered for the differentiation of causes of severe LVH. Due to the low number of patients included, we did not find a statistical significance for some typical target organ damages of AHT. This may be augmented by the large number of patients recently diagnosed with AHT. We only studied echocardiographies in patients with known AHT; therefore, no healthy controls were included. Moreover, we did not assess any longitudinal data. There were no echocardiographies available before the occurrence of AHT. On the other hand, there is a good comparability between the echocardiographic examinations of the patients, since only patients from our specialised clinic were included and almost all patients received had their echocardiography taken on the same machine and were supervised by the same two experienced cardiologists (TB, ASV). Due to the retrospective nature of the study, we did not have access to home BP measurements, which were not available in all patients at the same quality. Furthermore, we did not have any information on the presence of retinopathy in the majority of patients. 

An unanswered question is whether severe LVH is caused solely by AHT, or if there is an overlap with HCM. Further studies are needed to evaluate the usefulness of CMR and/or genetic testing in patients with severe LVH in the context of AHT. 

## 5. Conclusions

The present study shows a high prevalence of LV geometric abnormalities as well as severe LVH in the patients with AHT seen at a tertiary hypertension clinic, especially in those with uncontrolled AHT. The Sokolow-Lyon index is insufficient to detect LVH, but lateral T-wave inversions are relatively common in these patients. We were able to show that patients with severe LVH have more severe forms of AHT. The absence of other target organ damages or of secondary forms or refractory forms of AHT should raise the suspicion of aetiologies outside of AHT for the LVH.

## Figures and Tables

**Table 1:** Baseline characteristics.

Parameter	Total	Normal LV Geometry	Concentric Remodelling	Concentric Hypertrophy	Eccentric Hypertrophy	Severe LVH
	*n* = 400	*n* = 189	*n* = 125	*n* = 37	*n* = 20	*n* = 29
**General characteristics:**						
Female gender; *n* (%)	177 (44.3)	97 (51.3)	48 (38.4)	18 (48.6)	7 (35.0)	7 (24.1)
Male gender; *n* (%)	223 (55.7)	92 (48.7)	77 (61.6)	19 (51.4)	13 (65.0)	22 (75.9)
Age (years); median (IQR)	56 (43–70)	50.4 (36.5–60.8)	59.4 (47.9–72.1)	62.6 (53.7–74.3)	71.5 (61.5–77.6)	64.9 (52.9–73.7)
BMI (kg/m^2^); median (IQR)	27 (24–31)	27 (25–31)	26 (23–30)	28 (26–31)	27 (24–31)	29 (27–36)
**Hypertension:**						
Duration AHT (months); median (IQR)	23 (2–95)	13.1 (1.4–65.9)	34.1 (3.0–106.1)	27.7 (2.1–190.5)	73.6 (9.2–196)	34.8 (1.9– 115.4)
Secondary form of AHT; *n* (%)	130/349 (37.2)	49/166 (29.5)	40/108 (37.0)	19/33 (57.6)	7/17 (41.2)	15/25 (60.0)
**Mean ABPM results:**						
Systolic 24 h BP (mmHg); median (IQR)	133 (125–143)	132 (124–140)	131 (124–142)	142 (129–149)	136 (121–149)	145 (131–162)
Diastolic 24 h BP (mmHg); median (IQR)	81 (75–88)	81 (75–88)	81 (74–88)	80 (74–90)	76 (70–84)	85 (80–99)
Systolic awake BP (mmHg); median (IQR)	136 (127–146)	136 (127–144)	136 (126–144)	143 (132–153)	138 (124–147)	150 (135–162)
Diastolic awake BP (mmHg); median (IQR)	83 (77–91)	84 (77–91)	84 (76–91)	81 (77–91)	79 (71–87)	88 (82–100)
Systolic asleep BP (mmHg); median (IQR)	124 (115–138)	122 (114–133)	123 (115–134)	137 (125–144)	126 (111–146)	140 (125–155)
Diastolic asleep BP (mmHg); median (IQR)	73 (67–82)	72 (67–81)	73 (67–85)	78 (69–82)	71 (63–80)	81 (75–93)
Systolic 24 h mean BP ≥ 130 mmHg; *n* (%)	227 (56.8)	102 (55.7)	66 (54.1)	26 (72.2)	11 (61.1)	22 (75.9)
Diastolic 24 h mean BP ≥ 80 mmHg; *n* (%)	219 (54.8)	101 (55.2)	68 (55.7)	20 (55.6)	8 (44.4)	22 (75.9)
Systolic awake BP ≥ 135 mmHg; *n* (%)	219 (56.3)	96 (52.2)	66 (54.1)	25 (69.4)	10 (55.6)	22 (75.9)
Diastolic awake BP ≥85 mmHg; *n* (%)	177 (45.5)	86 (46.7)	54 (44.3)	14 (38.9)	5 (27.8)	18 (62.1)
Systolic asleep BP ≥ 120 mmHg; *n* (%)	244 (61.0)	104 (57.5)	73 (60.3)	30 (83.3)	12 (66.7)	25 (89.3)
Diastolic asleep BP ≥ 70 mmHg; *n* (%)	262 (65.5)	117 (64.6)	82 (67.8)	26 (72.2)	10 (55.6)	27 (96.4)
**Comorbidities:**						
Coronary artery disease; *n* (%)	85 (21.4)	36 (19.0)	24 (19.5)	10 (27.0)	4 (20.0)	11 (37.9)
Stroke/TIA; *n* (%)	45 (11.3)	19 (10.1)	12 (9.6)	6 (16.2)	2 (10.0)	6 (20.7)
Peripheral arterial disease; *n* (%)	25 (6.3)	9 (4.8)	8 (6.5)	3 (8.6)	3 (15.0)	2 (6.9)
Aortic aneurysm; *n* (%)	12 (9.8)	2/56 (3.6)	2/38 (5.3)	5/13 (38.5)	1/4 (25.0)	2/11 (18.2)
Diabetes; *n* (%)	70 (17.5)	25 (13.3)	25 (20.0)	9 (24.3)	5 (25.0)	6 (20.7)
HbA1c (%); median (IQR)	5.6 (5.3–5.9)	5.5 (5.2–5.8)	5.6 (5.3–5.9)	5.7 (5.4–5.9)	5.7 (5.4–6.5)	5.7 (5.2–6.0)
eGFR (ml/min/1.7); median (IQR)	84 (64–101)	93 (76–106)	82 (63–97)	64 (43.5–90)	81 (52–94)	59 (38–79)
Microalbuminuria ≥ 3.4 mg/mmol; *n* (%)	47/221 (21.3)	13/104 (12.5)	13/65 (20.0)	6/24 (25.0)	5/11 (45.5)	10/17 (58.8)
Active smokers; *n* (%)	80 (20.0)	38 (20.7)	31 (25.4)	4 (11.1)	5 (26.3)	2 (7.7)
History of smoking; *n* (%)	106 (27.4)	48 (26.1)	28 (23.0)	11 (30.6)	8 (42.1)	11 (42.3)
Family history HCM; *n* (%)	0 (0)	0 (0)	0 (0)	0 (0)	0 (0)	0 (0)
Family history SCD; *n* (%)	16/328 (4.9)	3/164 (1.8)	5/104 (4.8)	4/29 (13.8)	2/15.4 (15.4)	2/18 (11.1)
Family history AHT; *n* (%)	145/307 (44.9)	77/157 (49.0)	42/93 (45.2)	12/26 (46.2)	6/14 (42.9)	8/17 (47.1)
**Medication:**						
ACE/ARB; *n* (%)	286 (71.9)	124 (66.0)	92 (73.6)	28 (75.7)	19 (100)	23 (79.3)
CCB; *n* (%)	228 (57.0)	100 (52.9)	69 (55.2)	29 (78.4)	11 (55.0)	19 (65.5)
Diuretics; *n* (%)	130 (32.7)	37 (19.7)	39 (31.5)	24 (64.9)	11 (55.0)	19 (65.5)
Beta blockers; *n* (%)	132 (33.0)	50 (26.5)	34 (27.2)	20 (54.1)	12 (60.0)	16 (55.2)
Any antihypertensive; *n* (%)	348 (87.7)	156 (83.0)	110 (88.7)	35 (94.6)	19 (100.0)	28 (96.6)
Number of first line drugs (ACE/ARB/CCB/Diuretics); median (IQR)	2 (1–2)	1 (1–2)	2 (1–2)	2 (2–3)	2 (2–3)	2 (2–3)
Monotherapy (ACE/ARB/CCB/Diuretics); *n* (%)	113 (28.2)	60 (32.3)	40 (32.3)	5 (13.5)	4 (21.1)	4 (13.8)
Dual therapy (ACE/ARB/CCB/Diuretics); *n* (%)	132 (33.0)	59 (31.2)	41 (32.8)	14 (37.8)	8 (40.0)	9 (31.0)
Triple therapy (ACE/ARB/CCB/Diuretics); *n* (%)	87 (21.8)	27 (14.3)	26 (20.8)	16 (43.2)	7 (35.0)	13 (44.8)
**ECG:**						
Positive Sokolow-Lyon index; *n* (%)	16/360 (4.4)	4 (2.4)	4 (3.7)	2 (5.6)	3 (15.0)	3 (11.1)
Any ST-Depression; *n* (%)	55/359 (15.3)	7 (4.1)	10 (9.3)	6 (16.7)	3 (15.0)	5 (18.5)
Negative T V4-V6 in at least 1 lead; *n* (%)	31 (7.8)	18 (10.7)	14 (13.1)	6 (16.7)	10 (50.0)	7 (25.9)
**TTE:**						
Left ventricular ejection fraction (%); median (IQR)	61 (58–65)	61 (59–65)	62 (59–67)	63 (59–67)	56 (46–60)	56 (52–61)
Left atrial volume index (mL/m^2^); median (IQR)	29 (24–36)	27 (23–33)	28 (24–33)	32 (29–41)	34 (27–48)	37 (27–45)

LV: left ventricular; LVH: left ventricular hypertrophy; IQR: inter-quartile range; BMI: body-mass-index; AHT: arterial hypertension; ABPM: ambulatory blood pressure monitoring; BP: blood pressure; TIA: transient ischemic attack; eGFR: estimated glomerular filtration rate (CKD-EPI); SCD: sudden cardiac death; ACE: angiotensin converting enzyme inhibitor; ARB: angiotensin receptor blocker; CCB: calcium channel blocker; index; ECG: electrocardiogram; TTE: transthoracic echocardiography.

**Table 2:** Differences in patients with severe left ventricular hypertrophy (LVH) in comparison to patients with normal geometry, and concentric or eccentric hypertrophy, according to left ventricular geometry.

Parameter	Severe LVH *n* = 29	No LVH *n* = 189	LVH *n* = 57	Severe LVH vs. No LVH; *p*-Value	Severe LVH vs. LVH; *p*-Value
Female gender; *n* (%)	7 (24.1)	97 (51.3)	25 (43.9)	**0.009**	0.099
Age (years); median (IQR)	64.9 (52.9–73.7)	50.4 (36.5–60.8)	66.3 (55.1–75.4)	**0.001**	0.308
BMI (kg/m^2^); median (IQR)	29.0 (27.0–36.0)	27.0 (25.0–31.0)	27.0 (35.0–31.0)	**0.005**	**0.044**
Hypertension:					
Duration AHT (months); median (IQR)	34.8 (1.9–115.4)	13.1 (1.4–65.9)	39.9 (5.5–192.6)	0.307	0.740
Secondary form of hypertension	15/25 (60.0)	49/166 (29.5)	26/50 (52.0)	**0.005**	0.625
ABPM results:					
Systolic 24 h mean BP (mmHg); median (IQR)	145 (131–162)	132 (124–140)	141 (126–149)	**<0.0005**	0.056
Diastolic 24 h mean BP (mmHg); median (IQR)	85 (80–99)	81 (75–88)	80 (73–88)	**0.018**	**0.008**
Systolic awake BP (mmHg); median (IQR)	150 (135–162)	136 (127–144)	141 (129–150)	**<0.0005**	**0.035**
Diastolic awake BP (mmHg); median (IQR)	88 (82–100)	84 (77–91)	81 (74–90)	**0.034**	**0.008**
Systolic asleep BP (mmHg); median (IQR)	140 (125–155)	122 (114–133)	136 (120–144)	**<0.0005**	0.114
Diastolic asleep BP (mmHg); median (IQR)	81 (75–93)	72.0 (66.5–81.0)	76 (66–81)	**<0.0005**	**0.007**
Systolic 24 h mean BP ≥ 130 mmHg; *n* (%)	22 (75.9)	102 (55.7)	37 (68.5)	**0.044**	0.614
Diastolic 24 h mean BP ≥ 80 mmHg; *n* (%)	22 (75.9)	101 (55.2)	28 (51.9)	**0.043**	**0.037**
Comorbidities:					
Coronary heart disease; *n* (%)	11 (37.9)	36 (19.0)	14 (24.6)	**0.029**	0.217
Stroke/TIA; *n* (%)	6 (20.7)	19 (10.1)	8 (14.0)	0.116	0.539
Peripheral arterial disease; *n* (%)	2 (6.9)	9 (4.8)	6 (10.9)	0.646	0.708
Aortic aneurysm; *n* (%)	2/11 (18.2)	2/56 (3.6)	6/17 (35.3)	0.123	0.419
Diabetes; *n* (%)	6 (20.7)	25 (13.3)	14 (24.6)	0.268	0.791
eGFR (ml/min/1.7); median (IQR)	59.0 (38.0–79.0)	93.0 (75.8–106.0)	67.0 (47.0–90.0)	**<0.0005**	0.123
Microalbuminuria ≥ 3.4 mg/mmol; *n* (%)	10/17 (58.8)	13/104 (12.5)	11 (31.4)	**<0.0005**	0.076
Active or history of smoking; *n* (%)	13/26 (50.0)	86/184 (46.7)	28 (50.9)	0.835	1.000
Family history SCD; *n* (%)	2/18 (11.1)	3/164 (1.8)	6/42 (14.3)	0.077	1.000
Family history AHT; *n* (%)	8/17 (47.1)	77/157 (49.0)	18/40 (45.0)	1.000	1.000
Medication:					
ACE/ARB; *n* (%)	23 (79.3)	124 (66.0)	47 (83.9)	0.201	0.765
CCB; *n* (%)	19 (65.5)	100 (52.9)	40 (70.2)	0.234	0.806
Diuretics; *n* (%)	19 (65.5)	37 (19.7)	35 (61.4)	**<0.0005**	0.815
Beta blocker; *n* (%)	16 (55.2)	50 (26.5)	32 (56.1)	**0.004**	1.000
Any antihypertensive; *n* (%)	28 (96.6)	156 (83.0)	54 (96.4)	0.090	1.000
Monotherapy (ACE/ARB/CCB/Diuretics); *n* (%)	4 (13.8)	60 (32.3)	9 (16.1)	0.050	1.000
Dual therapy (ACE/ARB/CCB/Diuretics); *n* (%)	9 (31.0)	60 (32.3)	22 (39.3)	1.000	0.487
Triple therapy (ACE/ARB/CCB/Diuretics); *n* (%)	12 (41.4)	27 (14.5)	23 (41.1)	**0.004**	1.000
ECG:					
Positive Sokolow-Lyon index; *n* (%)	3 (11.1)	4 (2.4)	5 (8.9)	0.056	0.711
Negative T V4-V6 in at least 1 lead; *n* (%)	5 (18.5)	7 (4.1)	9 (16.1)	**0.014**	0.764
Any ST-Depression; *n* (%)	7 (25.9)	18 (10.7)	16 (28.6)	0.055	1.000
TTE:					
LVEF (%); median (IQR)	56 (52–61)	61 (59–65)	61 (56–66)	**<0.0005**	0.037
LAVI (mL/m^2^); median (IQR)	37 (27–45)	27 (23–33)	32 (28–46)	**0.002**	0.996

Severe left ventricular hypertrophy (LVH), defined as a septal thickness ≥ 15 mm; no LVH, defined as a relative wall thickness < 0.42 and a LVMI ≤ 115 g/m^2^ for men and ≤95 g/m^2^ for women; LVH, defined as an LVMI ≥ 115 g/m^2^ for men and ≥95 g/m^2^ for women, but septal thickness < 15 mm. IQR: inter-quartile range; BMI: body-mass-index; AHT: arterial hypertension; ABPM: ambulatory blood pressure monitoring; BP: blood pressure; TIA: transient ischemic attack; eGFR: estimated glomerular filtration rate (CKD-EPI); SCD: sudden cardiac death; ACE: angiotensin converting enzyme inhibitor; ARB: angiotensin receptor blocker; CCB: calcium channel blocker; LAVI: left atrial volume index; ECG: electrocardiogram. TTE: transthoracic echocardiography; LVEF: left ventricular ejection fraction; LAVI: left atrial volume index. Bold: statistically significant result.

## Data Availability

Data are not available due to ethical restrictions.

## Supplementary Materials

The following supporting information can be downloaded at: https://www.mdpi.com/article/10.3390/jcm12010228/s1, Figure S1: Post-hoc analyses from Kruskal-Wallis test with Dunn correction regarding systolic and diastolic blood pressure in the 24 h mean, awake and asleep values according to LV geometry and gender; Figure S2: Uncontrolled systolic or diastolic blood pressure according to LV geometry and gender.
