# Supplementary material for: Prevalence and Characterisation of Severe Left Ventricular Hypertrophy Diagnosed by Echocardiography in Hypertensive Patients
Source: J Clin Med. 2022 Dec 28;12(1):228. doi: 10.3390/jcm12010228 (PMC9821566; doi:10.3390/jcm12010228)
Supplement: Supplementary file 1 [file jcm-12-00228-s001.zip › jcm-2112070-supplementary.pdf]

Supplementary Materials: Prevalence and Characterisation of Severe Left Ventricular Hypertrophy Diagnosed by Echocardiography in Hypertensive Patients

Supplementary Figure S1:

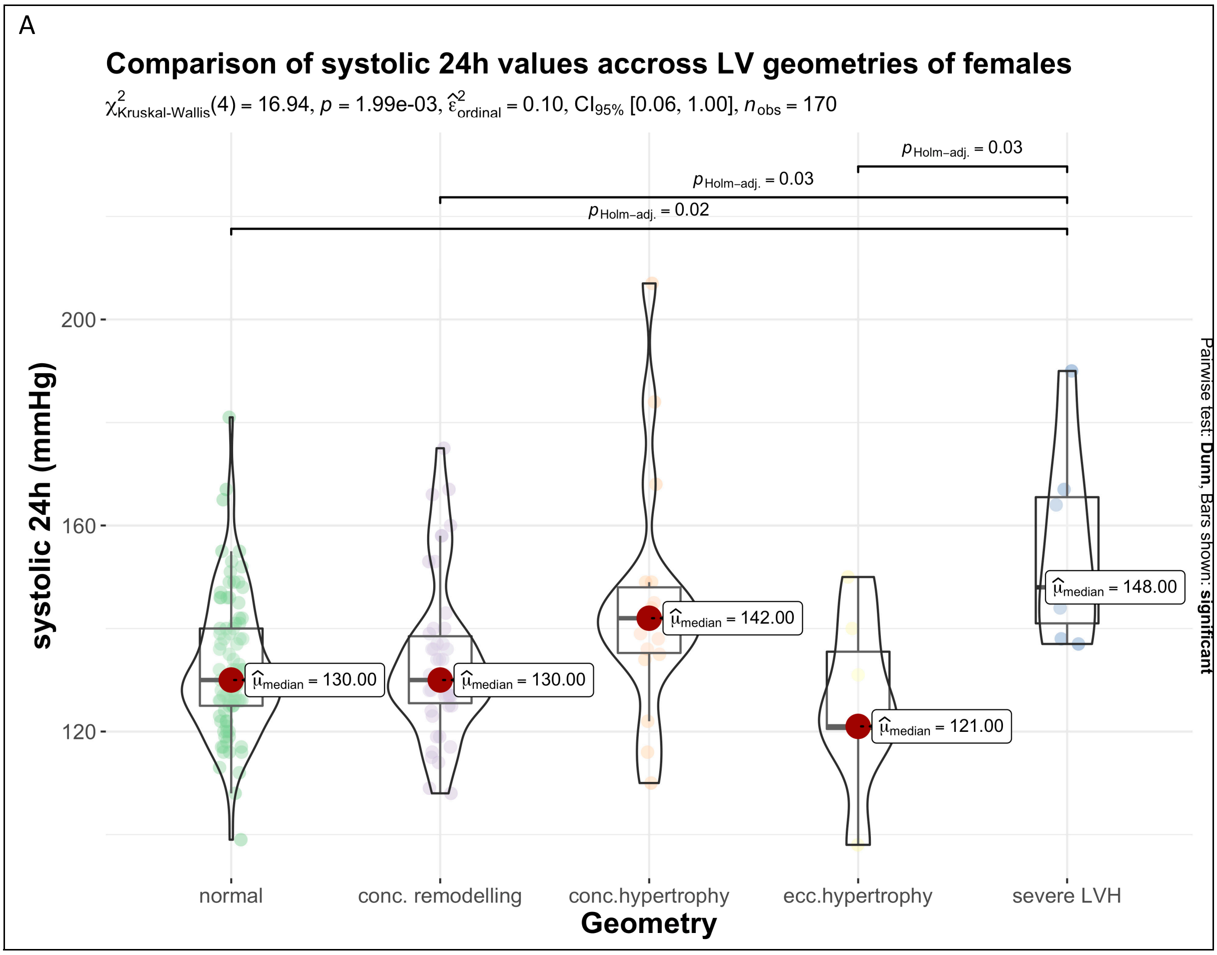

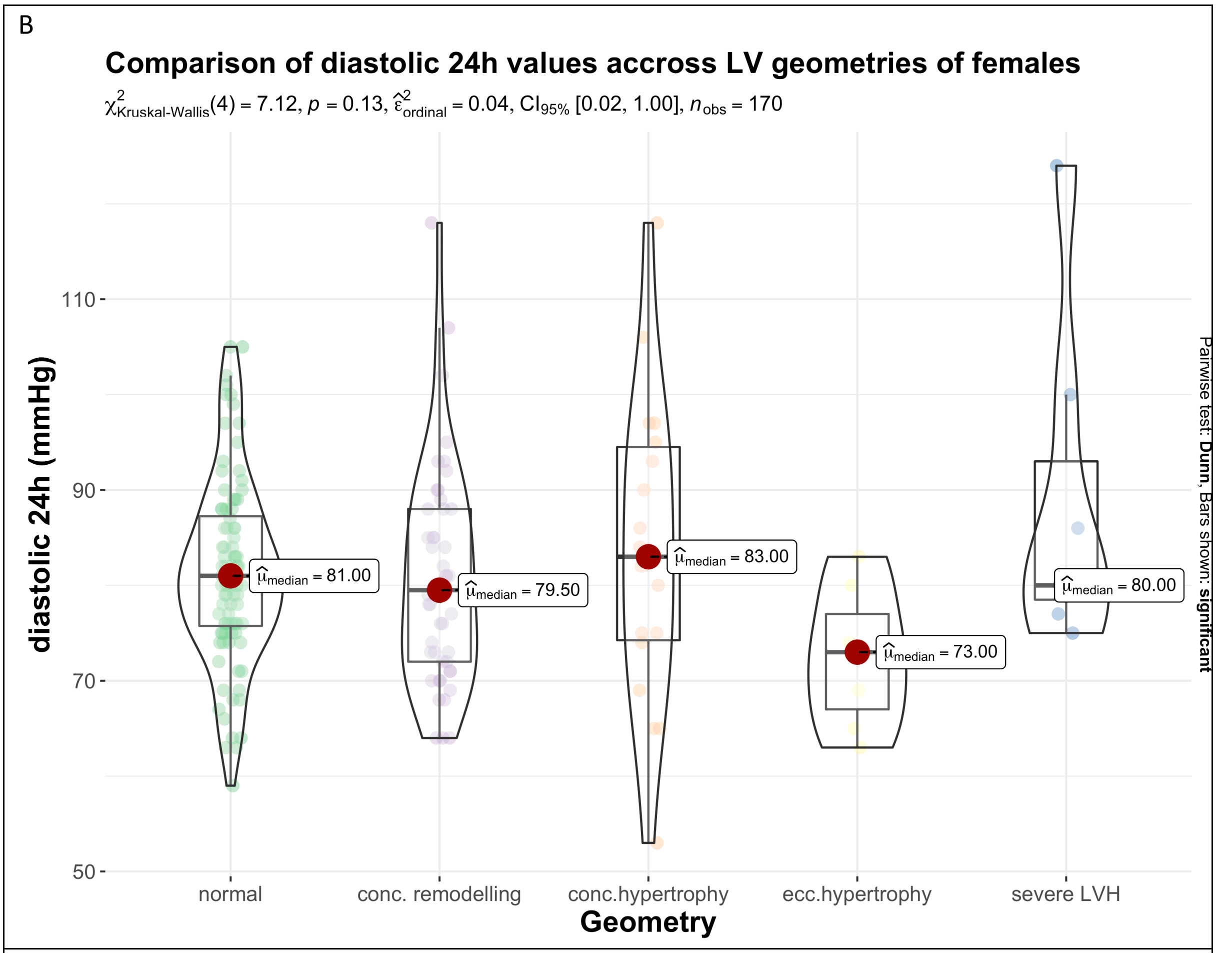
B
Comparison of diastolic 24h values accross LV geometries of females
$\chi^2_{\text{Kruskal-Wallis}}(4) = 7.12$, $p = 0.13$, $\hat{\varepsilon}^2_{\text{ordinal}} = 0.04$, $\text{CI}_{95\%}$ [0.02, 1.00], $n_{\text{obs}} = 170$
diastolic 24h (mmHg)
110
90
70
50
$\hat{\mu}_{\text{median}} = 81.00$
$\hat{\mu}_{\text{median}} = 79.50$
$\hat{\mu}_{\text{median}} = 83.00$
$\hat{\mu}_{\text{median}} = 73.00$
$\hat{\mu}_{\text{median}} = 80.00$
normal
conc. remodelling
conc.hypertrophy
ecc.hypertrophy
severe LVH
Geometry
Pairwise test: **Dunn**, Bars shown: **significant**

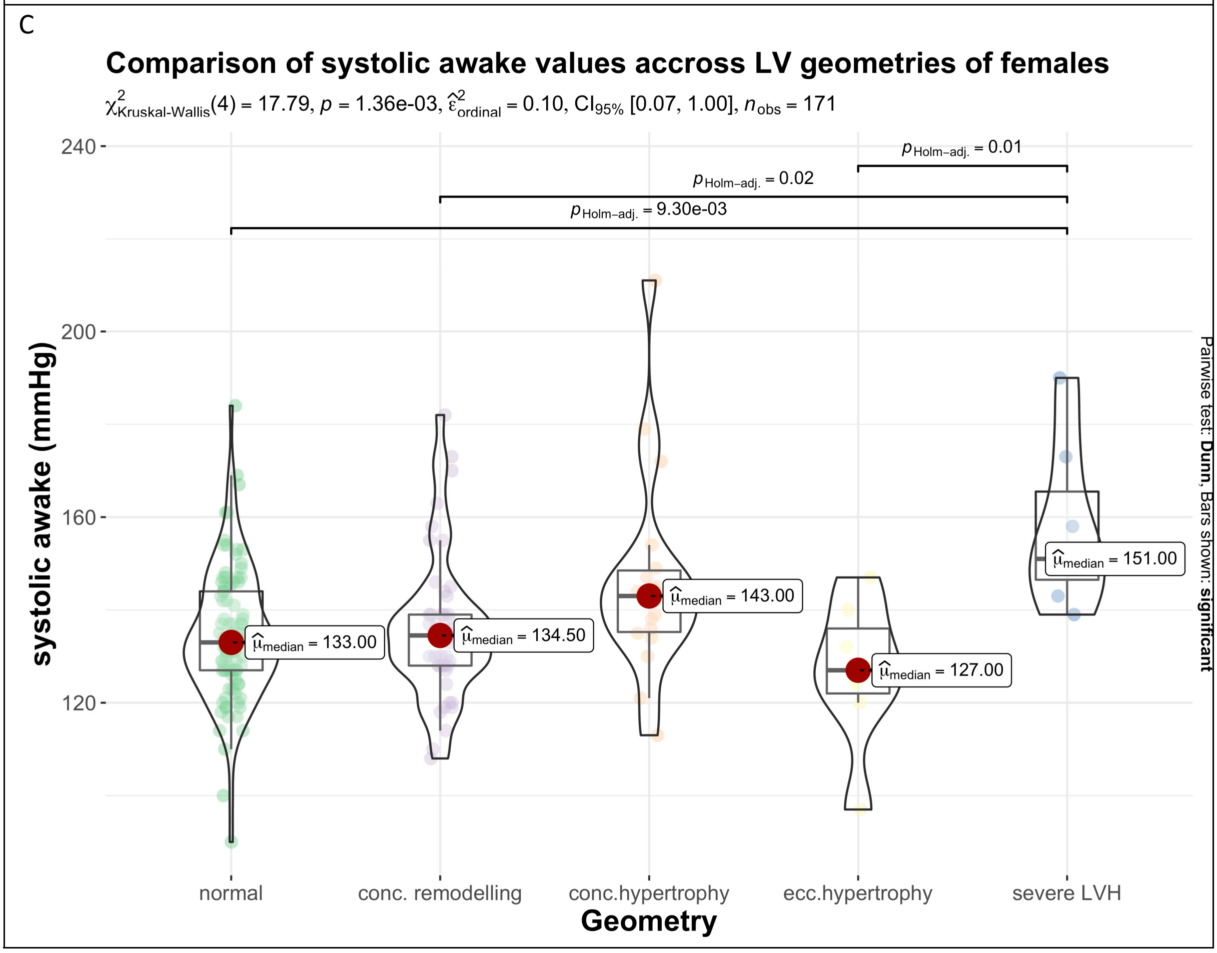
C
Comparison of systolic awake values accross LV geometries of females
$\chi^2_{\text{Kruskal-Wallis}}(4) = 17.79$, $p = 1.36\text{e-}03$, $\hat{\varepsilon}^2_{\text{ordinal}} = 0.10$, $\text{CI}_{95\%}$ [0.07, 1.00], $n_{\text{obs}} = 171$
systolic awake (mmHg)
240
200
160
120
$p_{\text{Holm-adj.}} = 0.01$
$p_{\text{Holm-adj.}} = 0.02$
$p_{\text{Holm-adj.}} = 9.30\text{e-}03$
$\hat{\mu}_{\text{median}} = 133.00$
$\hat{\mu}_{\text{median}} = 134.50$
$\hat{\mu}_{\text{median}} = 143.00$
$\hat{\mu}_{\text{median}} = 127.00$
$\hat{\mu}_{\text{median}} = 151.00$
normal
conc. remodelling
conc.hypertrophy
ecc.hypertrophy
severe LVH
Geometry
Pairwise test: **Dunn**, Bars shown: **significant**

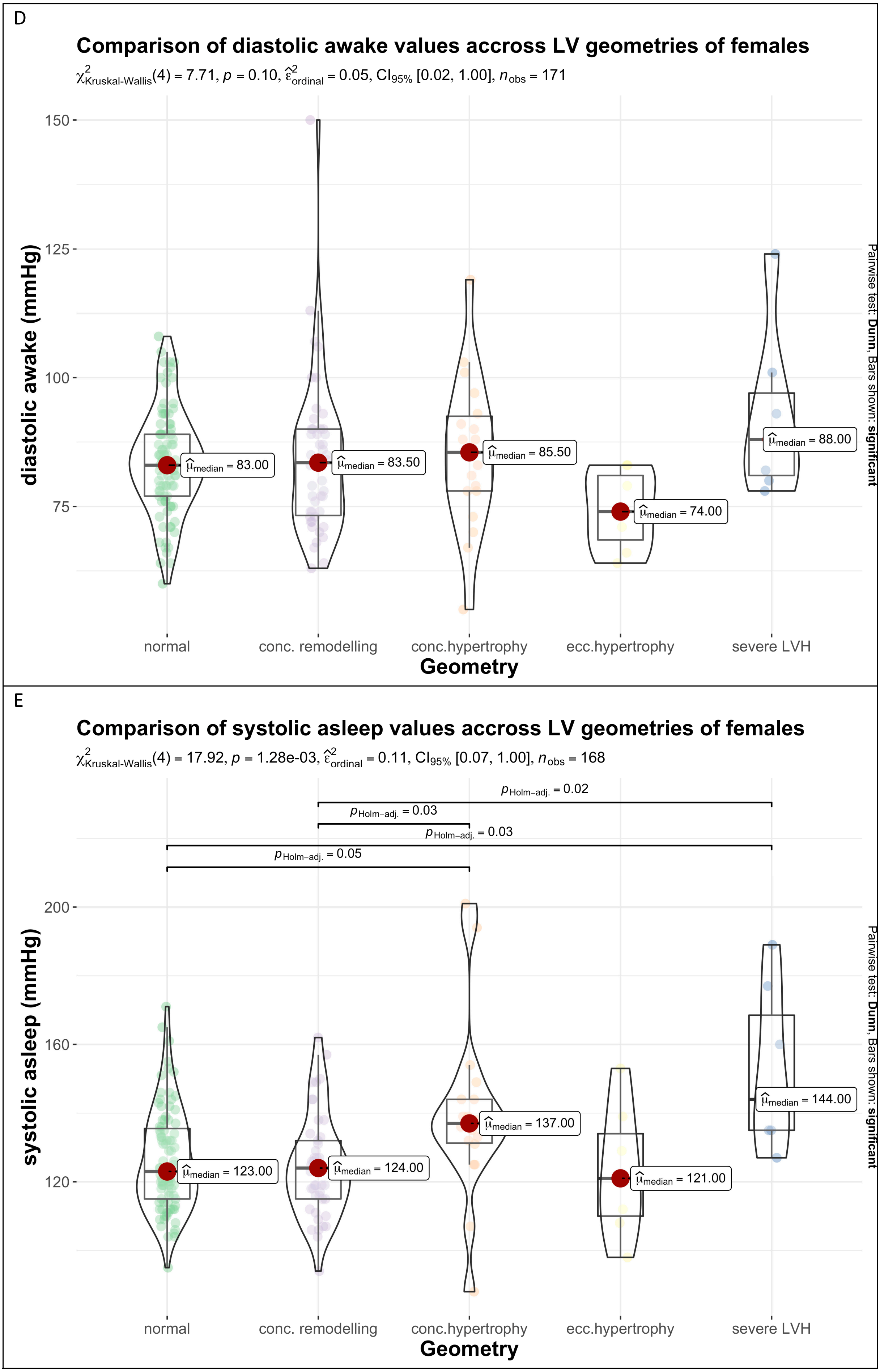

D
Comparison of diastolic awake values accross LV geometries of females
$\chi^2_{Kruskal-Wallis}(4) = 7.71$, $p = 0.10$, $\hat{\epsilon}^2_{ordinal} = 0.05$, $CI_{95\%}$ [0.02, 1.00], $n_{obs} = 171$
diastolic awake (mmHg)
150
125
100
75
$\hat{\mu}_{median} = 83.00$
$\hat{\mu}_{median} = 83.50$
$\hat{\mu}_{median} = 85.50$
$\hat{\mu}_{median} = 74.00$
$\hat{\mu}_{median} = 88.00$
normal
conc. remodelling
conc.hypertrophy
ecc.hypertrophy
severe LVH
Geometry
Pairwise test: Dunn, Bars shown: significant
E
Comparison of systolic asleep values accross LV geometries of females
$\chi^2_{Kruskal-Wallis}(4) = 17.92$, $p = 1.28e-03$, $\hat{\epsilon}^2_{ordinal} = 0.11$, $CI_{95\%}$ [0.07, 1.00], $n_{obs} = 168$
$p_{Holm-adj.} = 0.02$
$p_{Holm-adj.} = 0.03$
$p_{Holm-adj.} = 0.03$
$p_{Holm-adj.} = 0.05$
systolic asleep (mmHg)
200
160
120
$\hat{\mu}_{median} = 123.00$
$\hat{\mu}_{median} = 124.00$
$\hat{\mu}_{median} = 137.00$
$\hat{\mu}_{median} = 121.00$
$\hat{\mu}_{median} = 144.00$
normal
conc. remodelling
conc.hypertrophy
ecc.hypertrophy
severe LVH
Geometry
Pairwise test: Dunn, Bars shown: significant

F

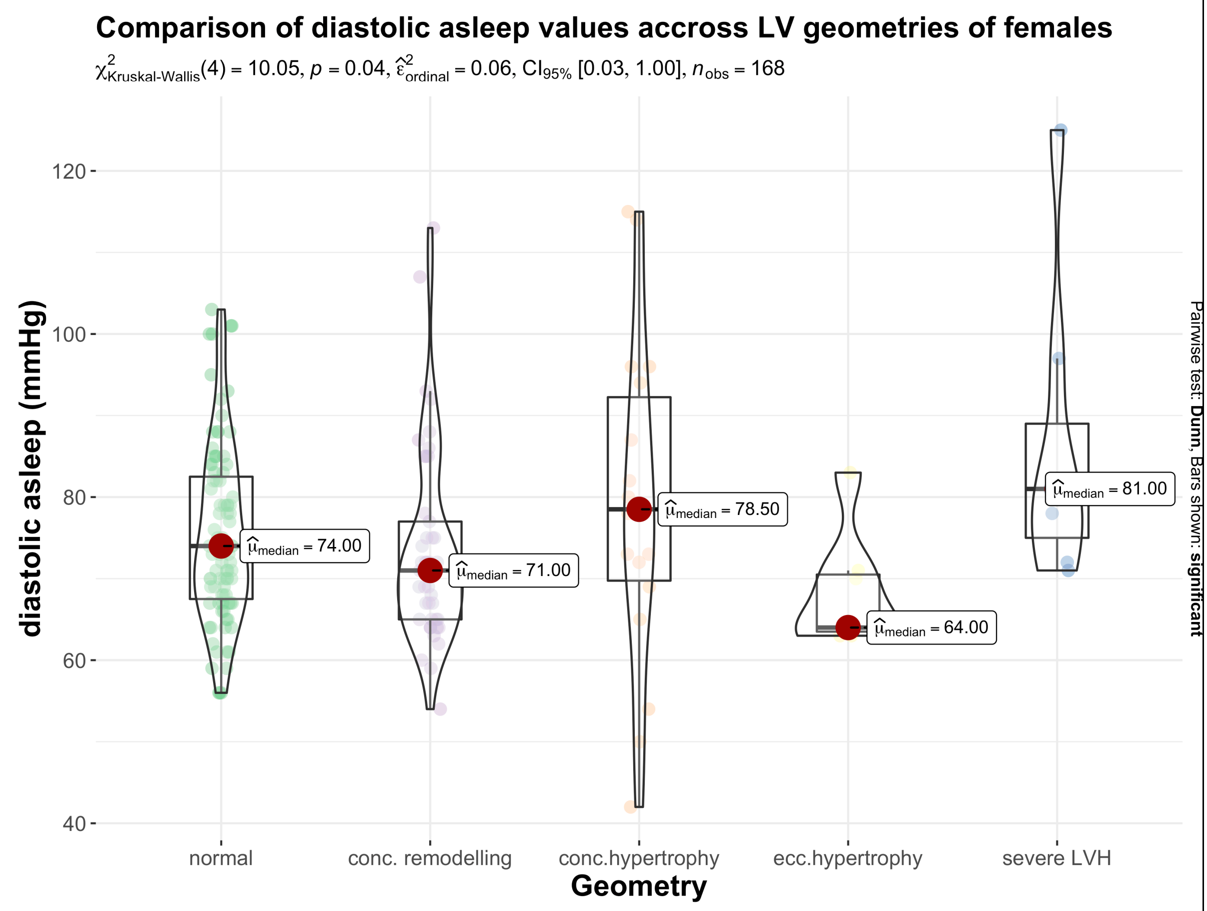

Comparison of diastolic asleep values accross LV geometries of females
χ²Kruskal-Wallis(4) = 10.05, p = 0.04, ε̂²ordinal = 0.06, CI95% [0.03, 1.00], nobs = 168
diastolic asleep (mmHg)
120
100
80
60
40
μ̂median = 74.00
μ̂median = 71.00
μ̂median = 78.50
μ̂median = 64.00
μ̂median = 81.00
normal
conc. remodelling
conc.hypertrophy
ecc.hypertrophy
severe LVH
Geometry
Pairwise test: Dunn, Bars shown: significant

G

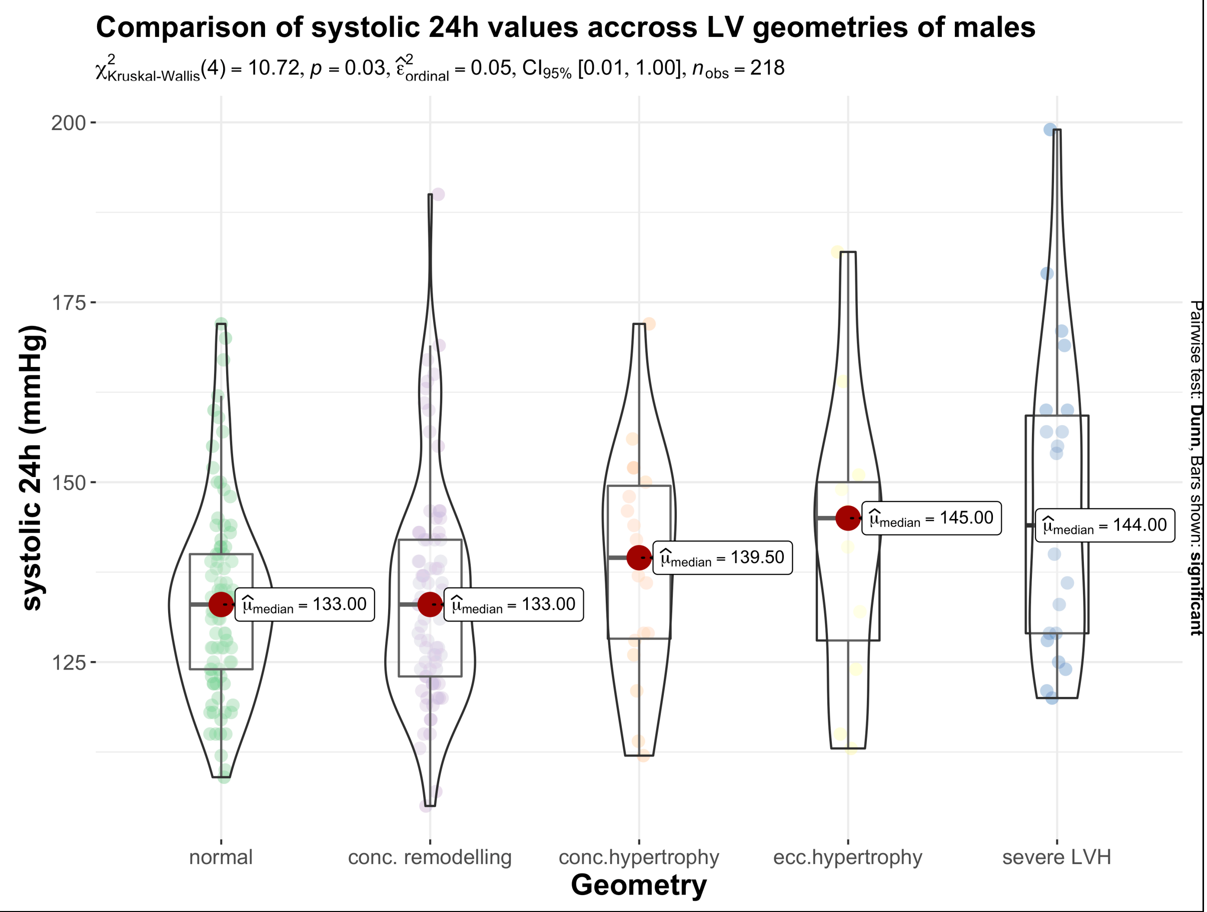

Comparison of systolic 24h values accross LV geometries of males
χ²Kruskal-Wallis(4) = 10.72, p = 0.03, ε̂²ordinal = 0.05, CI95% [0.01, 1.00], nobs = 218
systolic 24h (mmHg)
200
175
150
125
μ̂median = 133.00
μ̂median = 133.00
μ̂median = 139.50
μ̂median = 145.00
μ̂median = 144.00
normal
conc. remodelling
conc.hypertrophy
ecc.hypertrophy
severe LVH
Geometry
Pairwise test: Dunn, Bars shown: significant

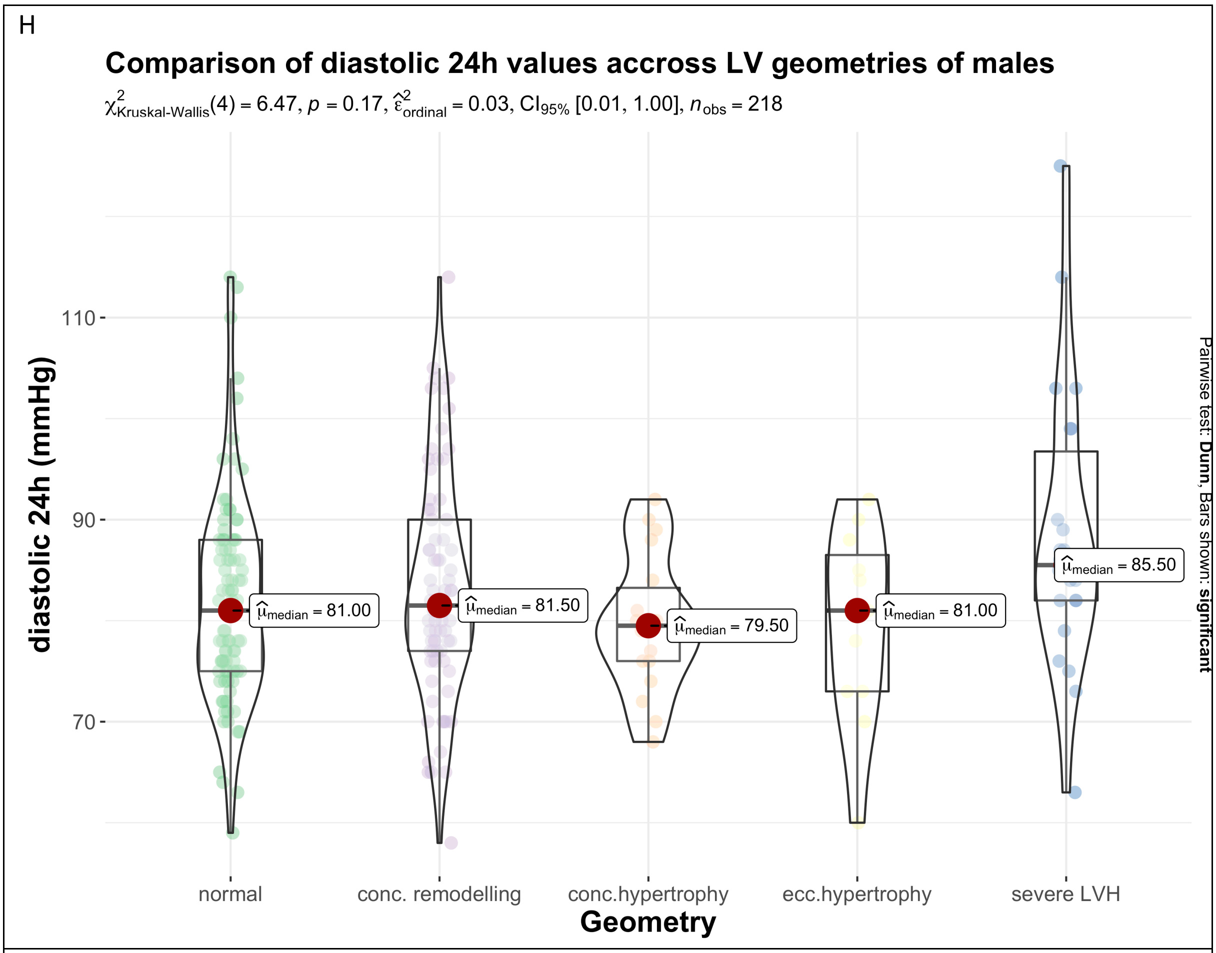
H
Comparison of diastolic 24h values accross LV geometries of males
$\chi^2_{\text{Kruskal-Wallis}}(4) = 6.47$, $p = 0.17$, $\hat{\varepsilon}^2_{\text{ordinal}} = 0.03$, $\text{CI}_{95\%}$ [0.01, 1.00], $n_{\text{obs}} = 218$
diastolic 24h (mmHg)
110
90
70
$\hat{\mu}_{\text{median}} = 81.00$
$\hat{\mu}_{\text{median}} = 81.50$
$\hat{\mu}_{\text{median}} = 79.50$
$\hat{\mu}_{\text{median}} = 81.00$
$\hat{\mu}_{\text{median}} = 85.50$
normal
conc. remodelling
conc.hypertrophy
ecc.hypertrophy
severe LVH
Geometry
Pairwise test: Dunn, Bars shown: significant

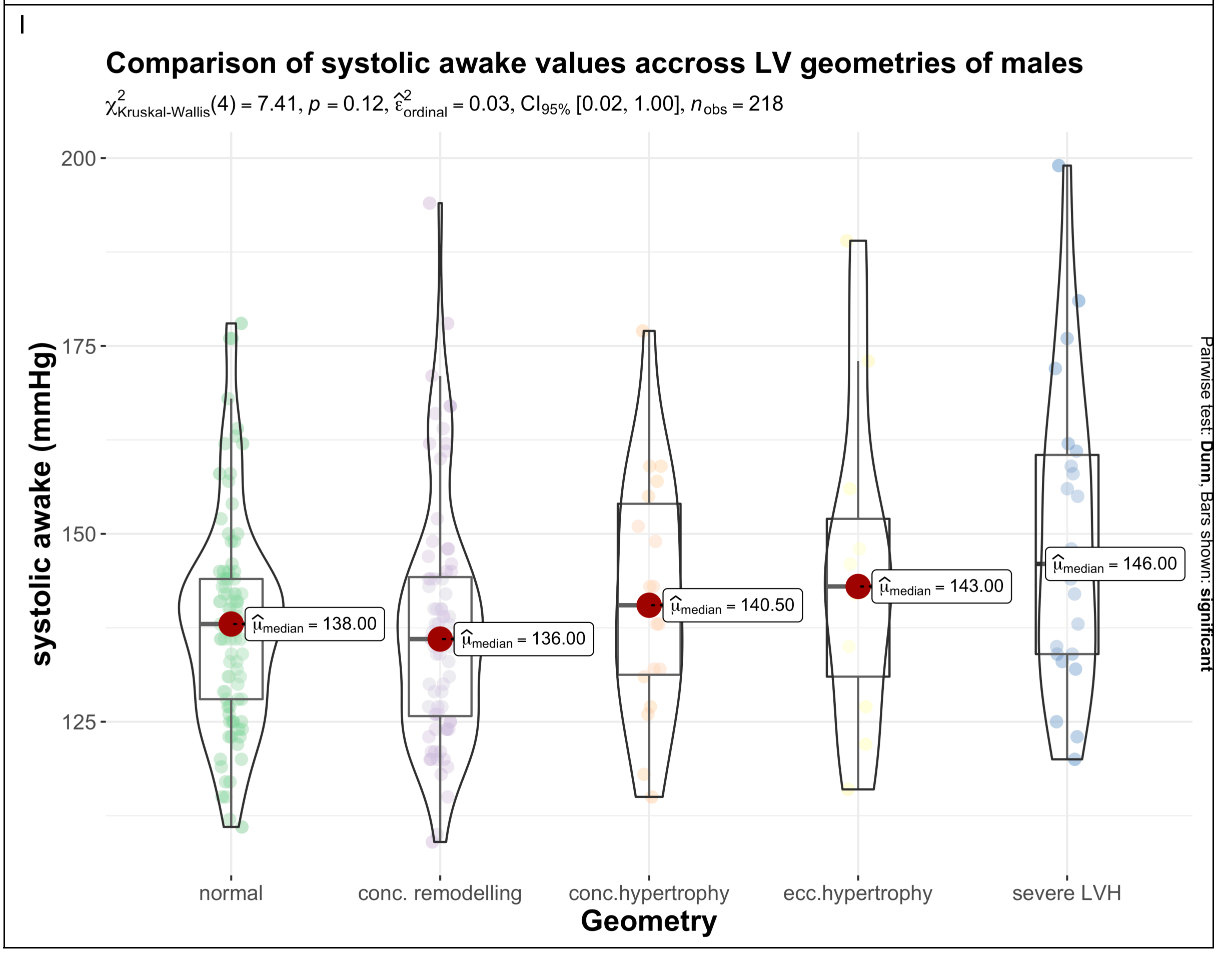
I
Comparison of systolic awake values accross LV geometries of males
$\chi^2_{\text{Kruskal-Wallis}}(4) = 7.41$, $p = 0.12$, $\hat{\varepsilon}^2_{\text{ordinal}} = 0.03$, $\text{CI}_{95\%}$ [0.02, 1.00], $n_{\text{obs}} = 218$
systolic awake (mmHg)
200
175
150
125
$\hat{\mu}_{\text{median}} = 138.00$
$\hat{\mu}_{\text{median}} = 136.00$
$\hat{\mu}_{\text{median}} = 140.50$
$\hat{\mu}_{\text{median}} = 143.00$
$\hat{\mu}_{\text{median}} = 146.00$
normal
conc. remodelling
conc.hypertrophy
ecc.hypertrophy
severe LVH
Geometry
Pairwise test: Dunn, Bars shown: significant

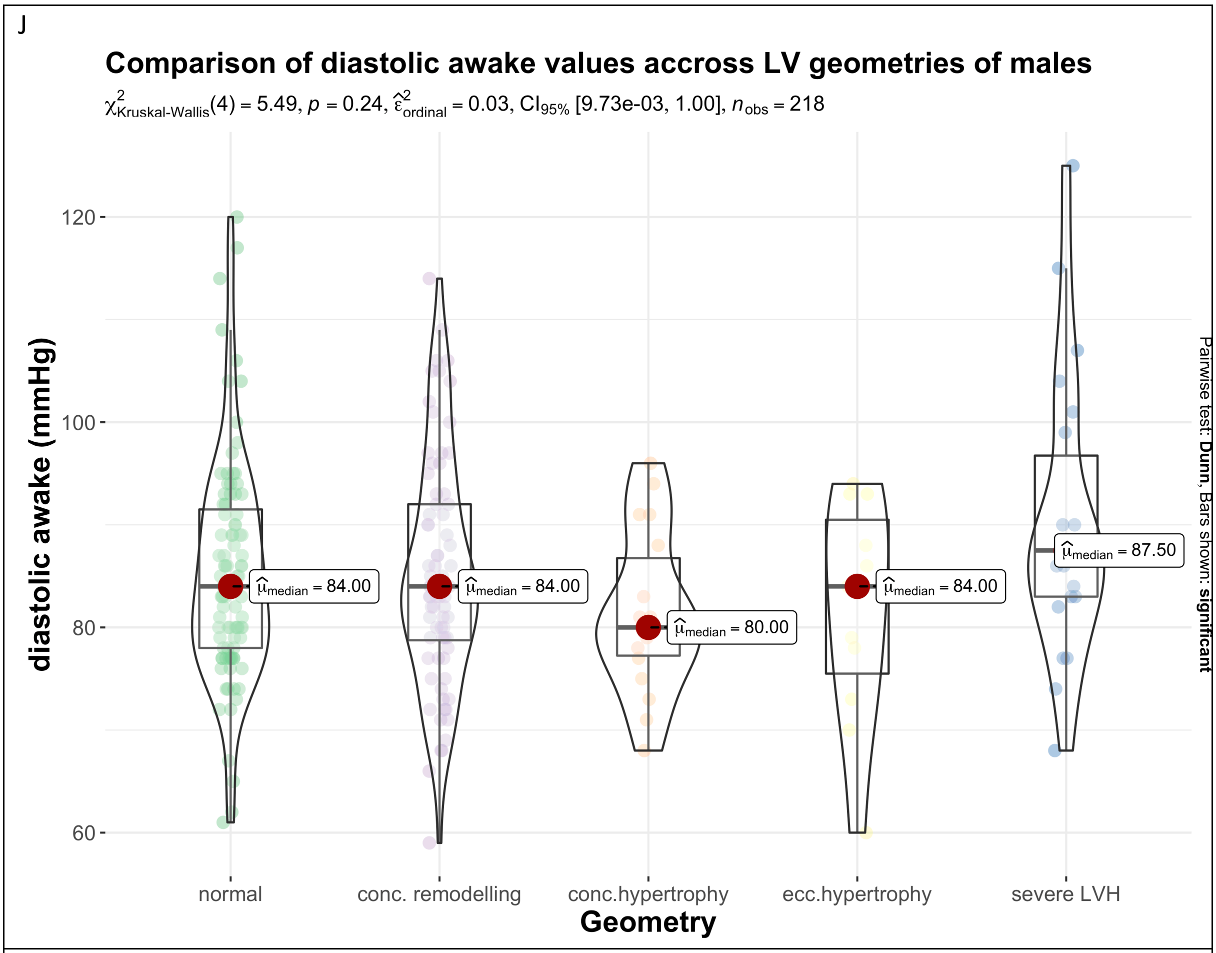

J
Comparison of diastolic awake values accross LV geometries of males
χ²Kruskal-Wallis(4) = 5.49, p = 0.24, ε̂²ordinal = 0.03, CI95% [9.73e-03, 1.00], nobs = 218
diastolic awake (mmHg)
120
100
80
60
μ̂median = 84.00
μ̂median = 84.00
μ̂median = 80.00
μ̂median = 84.00
μ̂median = 87.50
normal
conc. remodelling
conc.hypertrophy
ecc.hypertrophy
severe LVH
Geometry
Pairwise test: Dunn, Bars shown: significant

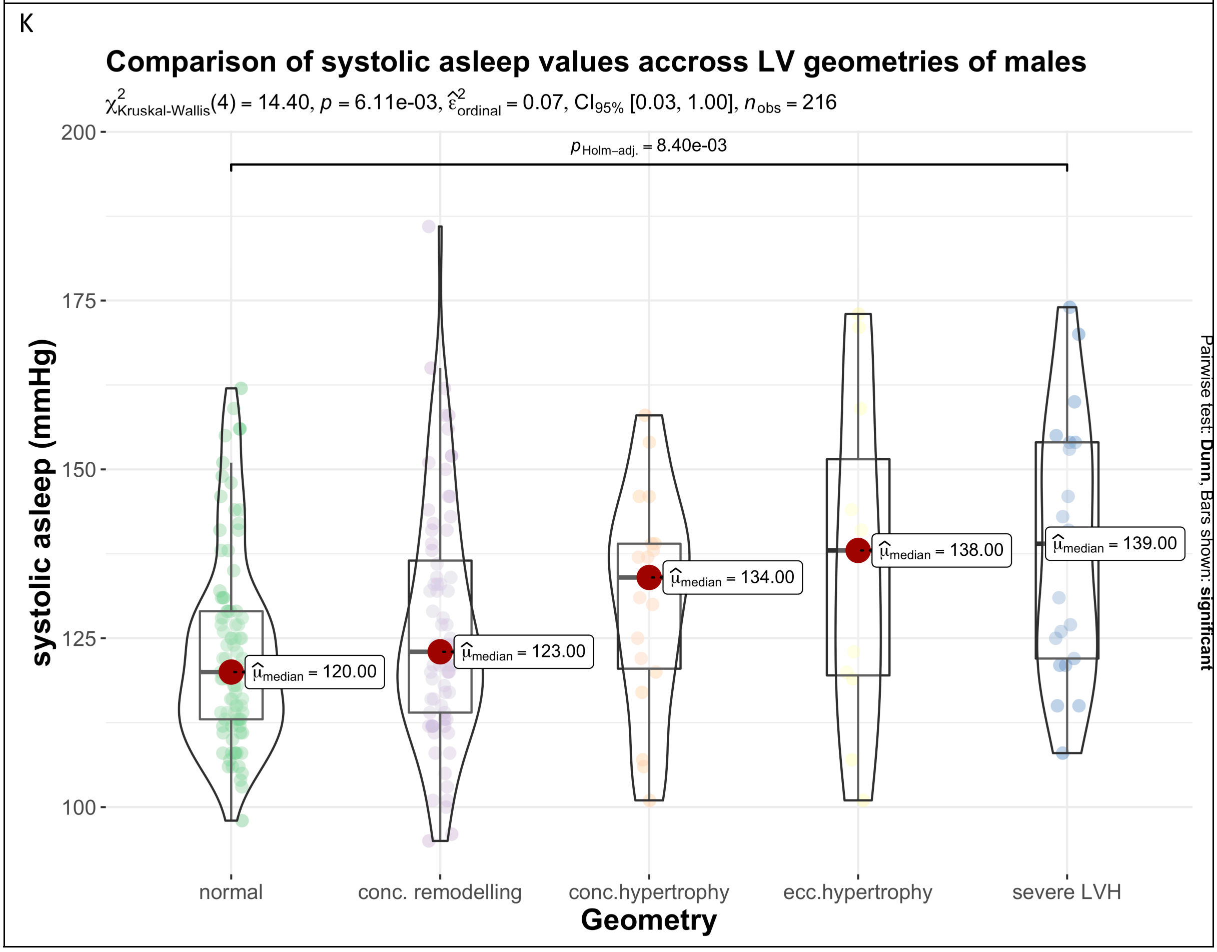

K
Comparison of systolic asleep values accross LV geometries of males
χ²Kruskal-Wallis(4) = 14.40, p = 6.11e-03, ε̂²ordinal = 0.07, CI95% [0.03, 1.00], nobs = 216
pHolm-adj. = 8.40e-03
systolic asleep (mmHg)
200
175
150
125
100
μ̂median = 120.00
μ̂median = 123.00
μ̂median = 134.00
μ̂median = 138.00
μ̂median = 139.00
normal
conc. remodelling
conc.hypertrophy
ecc.hypertrophy
severe LVH
Geometry
Pairwise test: Dunn, Bars shown: significant

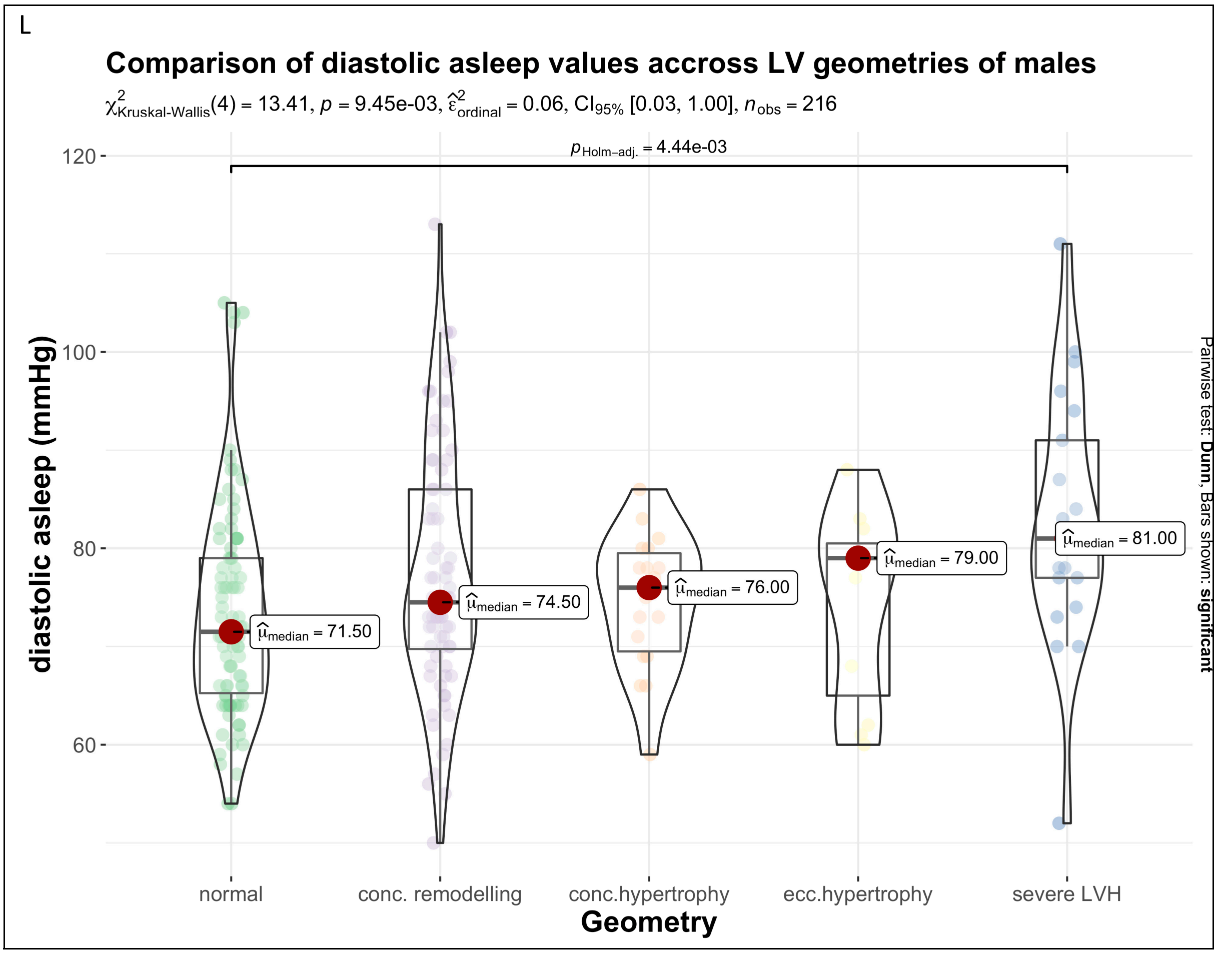

Figure S1. Post-hoc analyses from Kruskal-Wallis test with Dunn correction regarding systolic and diastolic blood pressure in the 24h mean, awake and asleep values according to LV geometry and gender. Panels A – F: female patients, panels G – L: male patients. Panels A and G: systolic 24h values, panels B and H: diastolic 24h values, panels C and I: systolic awake values, panels D and J: diastolic awake values, panels E and K: systolic asleep values, panels F and L: diastolic asleep values. Bars and p-values show significant differences.

Supplementary Figure S2:

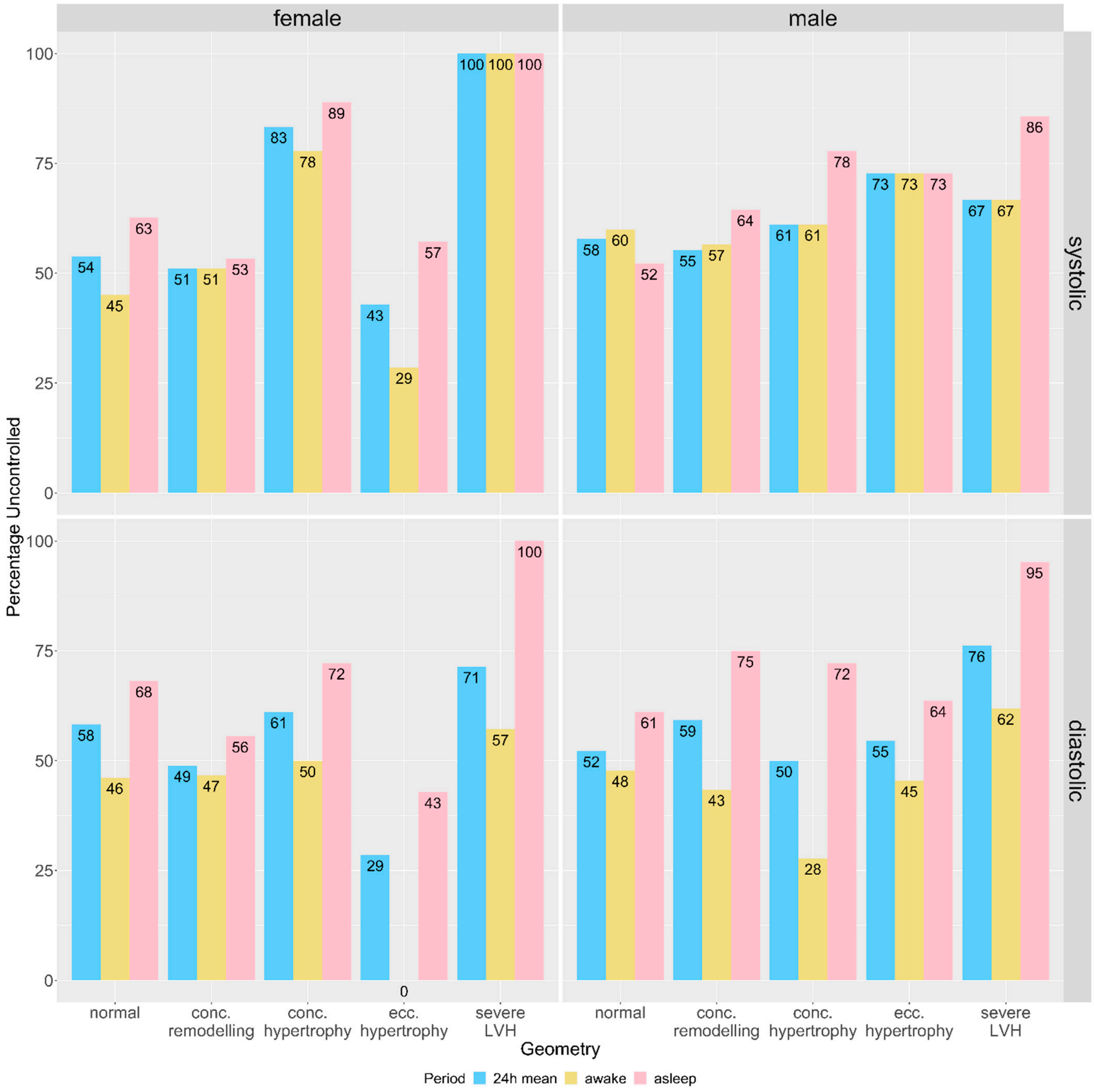

Figure S2. Uncontrolled systolic or diastolic blood pressure according to LV geometry and gender. Blue: 24h mean, yellow: awake, pink: asleep values.
